# Weibull Regression and Machine Learning Survival Models: Methodology, Comparison, and Application to Biomedical Data Related to Cardiac Surgery

**DOI:** 10.3390/biology12030442

**Published:** 2023-03-13

**Authors:** Thalytta Cavalcante, Raydonal Ospina, Víctor Leiva, Xavier Cabezas, Carlos Martin-Barreiro

**Affiliations:** 1 Department of Statistics, CASTLab, Universidade Federal de Pernambuco, Recife 50670-901, Brazil; 2 Department of Statistics, IME, Universidade Federal da Bahia, Salvador 40170-110, Brazil; 3 School of Industrial Engineering, Pontificia Universidad Católica de Valparaíso, Valparaíso 2362807, Chile; 4 Centro de Estudios e Investigaciones Estadísticas, Escuela Superior Politécnica del Litoral, Guayaquil 090902, Ecuador; 5 Faculty of Natural Sciences and Mathematics, Escuela Superior Politécnica del Litoral, Guayaquil 090902, Ecuador; 6 Faculty of Engineering, Universidad Espíritu Santo, Samborondón 0901952, Ecuador

**Keywords:** binary trees, Harrell index, model diagnostics, non-normal regression, random forest, statistical software, survival statistical analysis, variable importance, Weibull model

## Abstract

**Simple Summary:**

This article proposes a comparative study between two models that can be used by researchers for the analysis of survival data: Weibull regression and random survival forest. The models are compared considering the error rate, the performance of the model through the Harrell C-index, and the identification of the relevant variables for survival prediction. A statistical analysis of a data set from the Heart Institute of the University of São Paulo, Brazil, has been carried out. The proposal has many applications in biology and medicine.

**Abstract:**

In this article, we propose a comparative study between two models that can be used by researchers for the analysis of survival data: (i) the Weibull regression model and (ii) the random survival forest (RSF) model. The models are compared considering the error rate, the performance of the model through the Harrell C-index, and the identification of the relevant variables for survival prediction. A statistical analysis of a data set from the Heart Institute of the University of São Paulo, Brazil, has been carried out. In the study, the length of stay of patients undergoing cardiac surgery, within the operating room, was used as the response variable. The obtained results show that the RSF model has less error rate for the training and testing data sets, at 23.55% and 20.31%, respectively, than the Weibull model, which has an error rate of 23.82%. Regarding the Harrell C-index, we obtain the values 0.76, 0.79, and 0.76, for the RSF and Weibull models, respectively. After the selection procedure, the Weibull model contains variables associated with the type of protocol and type of patient being statistically significant at 5%. The RSF model chooses age, type of patient, and type of protocol as relevant variables for prediction. We employ the randomForestSRC package of the R software to perform our data analysis and computational experiments. The proposal that we present has many applications in biology and medicine, which are discussed in the conclusions of this work.

## 1. Introduction

Cardiovascular diseases are associated with arrhythmia, blood vessel problems, heart failure, myocardial infarction, and stroke, among others. These diseases are among the leading causes of death in the world [1]. In 2019 [2], more than 17 million people died from cardiovascular diseases.

In the care of cardiovascular diseases, their timely and accurate detection, as well as the determination of the factors that produce them are of the utmost importance for the survival of patients. Survival data have as response variable the time until the occurrence of a specific event of interest, called survival time or death time. One characteristic of survival data is the censorship of the observations [3], which is the partial observation of the response. Censored data appear with a certain frequency, as it is only sometimes possible to expect the event of interest to occur for all elements under study. In general, we can classify censoring into three groups [4]: (i) right censoring, when the event occurs after the beginning of the study; (ii) left censoring, when it occurs before the beginning of the study; and (iii) interval censoring, when the exact time of the censoring is not known, but only the interval in which it occurred. The present study uses a data set with right censoring and its analysis is performed with an R package named randomForestSRC [5]. There are three mechanisms of right censoring: (a) type I, in which the study ends after a pre-established period; (b) type II, in which the study ends after a death has occurred in a pre-established number of individuals under analysis; and (c) random censoring, which happens when the individual leaves the study without the event of interest having occurred. Even though the censored observations are partial, they provide essential information. Therefore, discarding these data can lead to biased inferences [6]. The statistical techniques used for this type of data are known as survival analysis, where survival is a term generally employed in the medical field, while in industrial research it is known as reliability.

Machine learning techniques are reliable and efficient for predicting cardiovascular diseases as noted in [7,8,9,10,11]. Several machine learning algorithms were proposed during the last decade for forecasting cardiovascular diseases using different parameters, data sets, and approaches. Diverse machine learning models, such as decision trees, support vector machines, artificial neural networks, naive Bayes, and random forests (RF) were employed to diagnose cardiovascular diseases [12]. An alternative machine learning technique that has been used to analyze survival data is the random survival forest (RSF) method [13], which instead of building a single survival tree, creates several of them, each using a random sample of the data. This technique is known as bagging [14] and estimates the survival function. The method is entirely non-parametric, so it does not require distributional assumptions in the relationship of the explanatory variables (covariates) and the response variable [15]. This procedure leads to a more accurate prediction considering traditional survival methods. The RSF method is based on the RF technique introduced in [16].

The RF technique can be employed for categorical response variables, referred to as classification, or for a continuous response, referred to as regression. Likewise, the covariates can be categorical or continuous. Subsequently, the RSF method was developed in [13], which is used to analyze right-censored survival data. Then, the RSF method is an extension of the RF technique [17,18]. For the RSF method, uniform consistency was proved in [19] under general rules, bootstrapping [20], and random selection of variables. Applications and recent advances from the RF technique to genomic data were reviewed in [21], including prediction and classification, variable selection, genetic pathway analysis, genetic association and detection of epistasis, as well as unsupervised learning. A new approach to competing risks using the RF technique was presented in [22].

The RSF method was compared in [23] with the conditional inference forests proposed in [24] to solve the bias problem for variables with many possible recursive partitioning; see also [25]. In [26], the authors researched how valuable the space extension technique in survival analysis is, which was proposed for classification analysis so far. It comprises building an extended variable space and inserting new variables in the study from the random combination of two or more original variables.

Risk models to predict dyslipidemia were formulated in [27], which are characterized by high levels of lipids and fats in the blood. The authors used the RSF method, considering the complex relationship between the variables. For comparison, they utilized the Cox regression model. Additionally, the Harrell concordance index (C-index) was employed to compare the models.

The RSF method was used in [28] to analyze the time until the recurrence of breast cancer. The considered model characterizes the survival function between patients with and without breast cancer recurrences, showing a strong potential to help health professionals in the prognosis, treatment, and decision-making of such conditions. Five models were fitted in [29], with four of them using the RSF method. In that study, the Cox model [30] was used for comparison. They utilized the following criteria: the Harrell C-index [31], and the Brier score index to compare models. The best-fitting model for prediction contained all covariates under RSF modeling. In this work, we adopt the Weibull model because is more flexible than the Cox model, as it allows for varying hazard rates over time and can handle different types of censoring. In addition, the Weibull model provides a more complete analysis due to its different types of hazard rates can help to state the survival distribution more accurately [32,33].

The main objective of this work is to compare the Weibull regression and RSF models for survival data analysis using three criteria: the error rate, C-index, and identification of the most relevant variables for survival prediction. For the computational experiments, we used a data set that studies the length of stay (in hours) of patients undergoing cardiac surgery inside the surgical ward, as a function of some covariates. We encourage researchers to utilize our methodology, which facilitates the analysis of survival data because it allows for choosing the best model, and therefore making the best prediction, according to the criteria selected by the data analyst. The article is organized as follows. Section 2, Section 3 and Section 4 present background on survival analysis, Weibull regression, and RSF models. In Section 5, we analyze the data, and Section 6 provides some discussion and conclusions.

## 2. Survival Analysis

In this section, we present background related to the Kaplan–Meier method, which is used to estimate the survival function. In addition, the Nelson–Aalen method, employed to estimate the cumulative risk function, is presented.

### 2.1. Kaplan–Meier Estimator

To estimate the survival function [34], denoted by S(t), in the presence of censored observations, we use the Kaplan–Meier method [35], also known as the product limit estimator. Let $t_{1}<\ldots <t_{k}$ be *k* observed and ordered times, $d_{j}$ be the number of deaths at $t_{j}$, and $n_{j}$ be the number of individuals at risk until $t_{j}$ (exclusive), that is, the individuals who survived and were not censored until the instant immediately before $t_{j}$, for j∈{1,…,k}. The Kaplan–Meier estimator is defined as $\hat{S}\left(t\right)=\prod_{j:t_{j}\leq t}\left(1-d_{j}/n_{j}\right)$. This estimator is an adaptation of the empirical survival function. It considers as time intervals as the number of distinct deaths exist, where the limits of the intervals are the death times in the sample.

Consider, under the null hypothesis ($H_{0}$), the equality of survival curves, where the alternative hypothesis ($H_{1}$) indicates that a difference between survival curves exists. To compare different survival curves, the Mantel log-rank test [36] is often used. Under $H_{0}$, the corresponding test statistic has a chi-square distribution with r−1 degree of freedom considering large samples, where *r* is the number of groups to be compared.

### 2.2. Nelson–Aalen Estimator

The risk function (or hazard/failure rate) is defined as the probability that the death occurs in the interval of time [t,t+Δt), where Δt is an infinitely small time in relation to *t*. However, with the cumulative risk function, denoted by H(t), we obtain the risk of an event occurring at all times up *t*, that is, the cumulative risk is the sum of all risks at all times up *t*.

The Nelson–Aalen estimator [37,38] is used to obtain the cumulative risk function. However, it can also be utilized for the survival function through a relationship stated as S(t)=exp(−H(t)) or equivalently H(t)=−log(S(t)). Therefore, the Nelson–Aalen estimator for the survival function is defined as $\tilde{S}\left(t\right)=\exp\left(-\hat{H}\left(t\right)\right)$, where $\hat{H}\left(t\right)=\sum_{j:t_{j}\leq t}\left(d_{j}/n_{j}\right)$ and $d_{j},n_{j}$ are defined as in the case of the Kaplan–Meier estimator [39].

## 3. Weibull Regression Model

In this section, the Weibull regression is formulated. For this regression model, the Weibull and extreme value distributions are necessary. The method of maximum likelihood that allows us to estimate the parameters of the model is also presented here. Then, the analysis of residuals for the Weibull regression is discussed.

### 3.1. Formulation

Let $T_{1},\ldots ,T_{n}$ be independent random variables that follow a Weibull distribution with parameters of shape γ≥0 and scale α≥0. Then, the probability density function (PDF) is given by $f\left(t,\alpha ,\gamma \right)=\alpha \gamma {\left(\alpha t\right)}^{\gamma -1}\exp\left(-{\left(\alpha t\right)}^{\gamma }\right)$, for t>0; for more details, see [40]. Assume that each $T_{i}$ depends on a vector with *p* covariates.

The standard extreme value distribution for a variable *Y* with scale parameter σ and location μ has a PDF given by f(y,μ,σ)=(1/σ)exp(−(x−μ)/σ)exp(−exp(−(x−μ)/σ))), for y∈R. If we are interested in determining the relationship between $T_{i}$ and a vector of covariates, we can make use a regression model. Then, we choose the Weibull regression [41], whose model is expressed as $Y_{i}=\log\left(T_{i}\right)=x_{i}^{⊤}\beta +\sigma \nu_{i}$, for i∈{1,…,n}, where $Y_{i}$ follows an extreme value distribution with scale parameter σ, and location $\mu_{i}=x_{i}^{⊤}\beta$; $x_{i}^{⊤}=\left(1,x_{i1},\ldots ,x_{ip}\right)$ is a vector with values of the covariates; $\beta ={\left(\beta_{0},\beta_{1},\ldots ,\beta_{p}\right)}^{⊤}$ is a vector of unknown regression parameters; and the model error $\nu_{i}=\log\left(\varepsilon_{i}\right)$ follows a standard extreme value distribution.

### 3.2. Point Estimation

Consider the pairs $\left(T_{1},\delta_{1}\right),\ldots ,\left(T_{n},\delta_{n}\right)$, where $T_{i}$ is the death or censoring time of individual *i*, and $\delta_{i}$ is a variable indicating death or censorship of this individual, that is, we assign $\delta_{i}=1$ if the individual *i* experienced a death, and $\delta_{i}=0$ for a censoring, with i∈{1,…,n}.

We use the maximum likelihood method to estimate the parameters of the Weibull regression model. Let $Y_{1}=\log\left(T_{1}\right),\ldots ,Y_{n}=\log\left(T_{n}\right)$ be independent random variables, such that $Y_{i}$ follows an extreme value distribution with scale parameter σ, location parameter $\mu_{i}=x_{i}^{⊤}\beta$, and $\theta ={\left(\beta^{⊤},\sigma \right)}^{⊤}$ is a parameter vector of dimension (p+2)×1 of unknown parameters to be estimated. The corresponding likelihood function considering right-censored data is expressed by $L\left(\theta \right)\propto \prod_{i=1}^{n}f{\left(y_{i},\theta \right)}^{\delta_{i}}S{\left(y_{i},\theta \right)}^{1-\delta_{i}}$, where $f\left(y_{i},\theta \right)=\left(1/\sigma \right)\exp\left(\left(\left(y_{i}-\mu_{i}\right)/\sigma \right)-\left(\left(y_{i}-\mu_{i}\right)/\sigma \right)\right)$ is the PDF, $\delta_{i}$ is the indicator variable of dead or censorship, and $S\left(y_{i},\theta \right)=\exp\left(-\exp\left(\left(y_{i}-\mu_{i}\right)/\sigma \right)\right)$ is the survival function. Recall $\delta_{i}=1$ if the individual experienced a death and $\delta_{i}=0$ otherwise. Note that the contribution of each uncensored observation is its PDF and that each censored observation contributes by means of the survival function. The maximum likelihood estimators of the regression coefficients and the scale parameter are solutions of the equations resulting from taking derivatives of the logarithm of L(θ). As these equations do not have a closed-form solution, we must obtain the maximum likelihood estimates of the unknown parameters employing numerical approximation methods.

### 3.3. Adequacy of the Fitted Model

Once a regression model is fitted to a data set, one needs to evaluate its fit utilizing validation and diagnostics. We make it through the analysis of the Cox–Snell, martingale, and deviance residuals.

The Cox–Snell residuals [42] are used to evaluate the global fit of the selected model. These residuals are defined as ${\hat{e}}_{i}=\hat{H}\left(t_{i}|x_{i}^{⊤}\right)=-\log\left(\hat{S}\left(t_{i}|x_{i}^{⊤}\right)\right)$, for i∈{1,…,n}, where $\hat{H}$ is the cumulative risk function obtained from the fitted model, and $\hat{S}$ is the estimated survival function. If there are few censored observations and exponential or Weibull models are being used, it is appropriate to adjust the censored residuals and treat them as uncensored. Therefore, for a given censored $t_{i}$, the Cox–Snell residual is given by

$${\hat{e}}_{i}=-\log\left(\hat{S}\left(t_{i}|x_{i}^{⊤}\right)\right)+1,\;i\in \left\{1,\ldots ,n\right\}.$$

For the Weibull regression model, the Cox–Snell residuals are given by

$${\hat{e}}_{i}=\left\{\begin{array}{cc} {\left(t_{i}\exp\left(-{\hat{\mu }}_{i}\right)\right)}^{1/\hat{\sigma }}, & \mathrm{if}\delta =1;\\ {\left(t_{i}\exp\left(-{\hat{\mu }}_{i}\right)\right)}^{1/\hat{\sigma }}+1, & \mathrm{if}\delta =0; \end{array}\right.$$

where $1/\hat{\sigma }=\hat{\gamma }$ and i∈{1,…,n}. If the fitted model is suitable for the data, then the Cox–Snell residuals must follow a standard exponential distribution. In this context, we can use the graph of the survival curves of the residuals obtained by the Kaplan–Meier estimator and the standard exponential model. As the curves are closer, the model is better fitted.

The martingale residuals are asymmetric and take a maximum value of 1 and a minimum at −∞. The martingale residuals are defined as

$${\hat{m}}_{i}=\delta_{i}-{\hat{e}}_{i},\;i\in \left\{1,\ldots ,n\right\},$$

where $\delta_{i}$ is the death or censoring variable, and ${\hat{e}}_{i}$ are the Cox–Snell residuals. Note that the martingale residuals for censored observations assume negative values. Therefore, for the Weibull regression model, these residuals take the form stated as

$${\hat{m}}_{i}=\delta_{i}-{\left(t_{i}\exp\left(-{\hat{\mu }}_{i}\right)\right)}^{1/\hat{\sigma }},\;i\in \left\{1,\ldots ,n\right\}.$$

The martingale residuals can be seen as an estimate of the number of deaths observed in the data but not predicted by the model. They are used to examine the best functional form (linear or nonlinear) for a given variable in a regression model. Furthermore, they can identify outliers in the dataset. However, it is generally better to employ deviance residuals.

The deviance residuals are transformations from the martingale residuals to mitigate the asymmetry. In general, this facilitates the detection of atypical points (outliers). The deviance residuals are defined as

$${\hat{d}}_{i}=\mathrm{sign}\left({\hat{m}}_{i}\right){\left(-2\left({\hat{m}}_{i}+\delta_{i}\log\left(\delta_{i}-{\hat{m}}_{i}\right)\right)\right)}^{1/2},\;i\in \left\{1,\ldots ,n\right\},$$

where ${\hat{m}}_{i}$ is the martingale residual, with the deviance residual having a random behavior around zero.

## 4. Random Survival Forest Method

In this section, some aspects related to the RSF method are discussed. The steps of this method are explained in algorithmic form and a flow diagram is also included. Due to the use of a binary tree, node splitting and prediction are mentioned. The cumulative hazard function for the out-of-bag (OOB) set is described. The variable importance (VIMP) in the prediction and its error are also established here. This section ends showing a flow diagram that details the computation of the Harrell C-index.

### 4.1. Algorithm

Similar to the classification and regression trees [43], survival trees are binary and grow recursively splitting nodes, denoted generically by *h*. A tree grows starting at the root node, which is the top of the tree and contains all the data. Using a separation rule to split the space of variables, the root node is split into two child nodes: to the left and right. Furthermore, each one of them is also split into new child nodes. The process is repeated recursively for each subsequent node. The most extreme nodes in a tree are called terminal nodes. A proposed splitting rule at node *h* in a given variable *x* is always of the form x≤c and x>c, where *c* is a threshold value.

**Figure 1:**
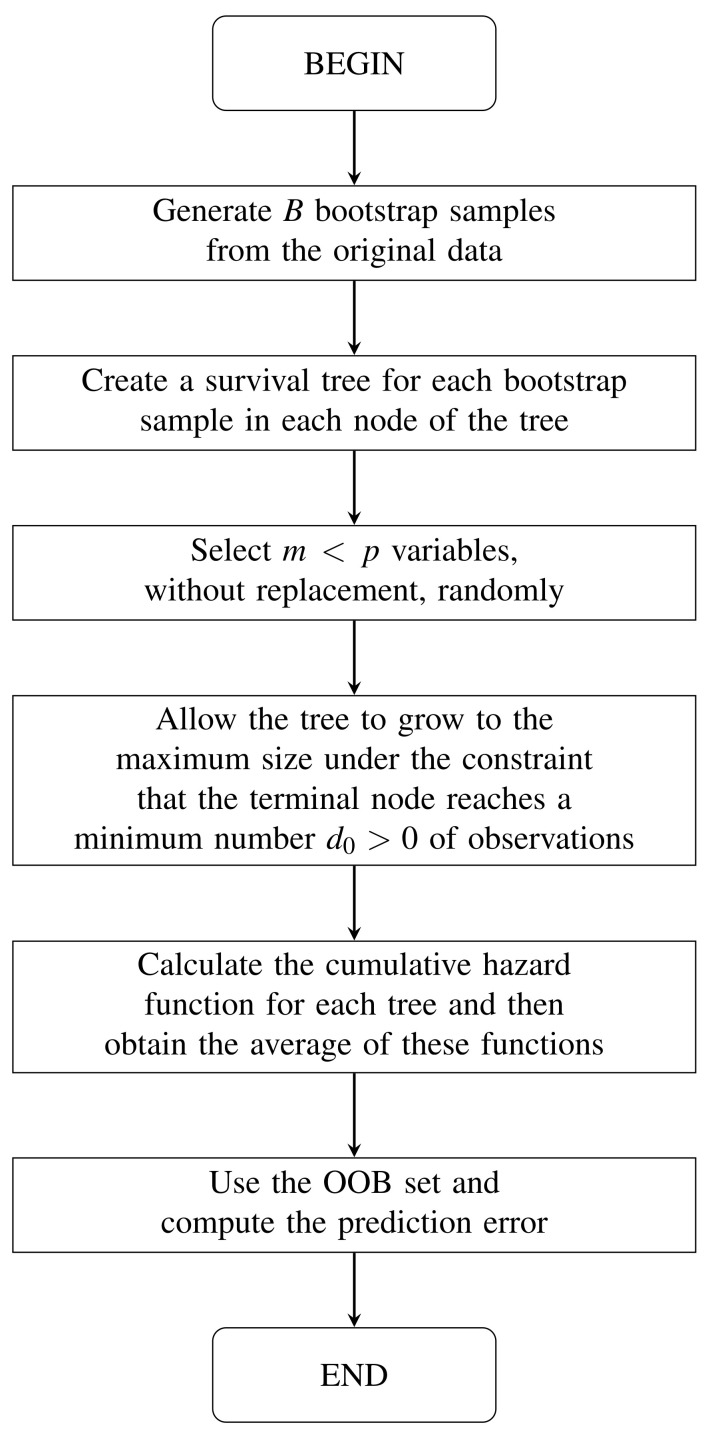
Flow diagram of the random survival forest method.

In Algorithm 1, we can see the RSF method [13]; see also Figure 1. Both the algorithm and the figure show the steps of the RSF method. We include both for the benefit of different types of readers. For some of them, such as developers, the algorithm may be more convenient. For other readers, it may be easier to understand the steps if they are shown in a flowchart.
**Algorithm 1:** Random survival forest method.
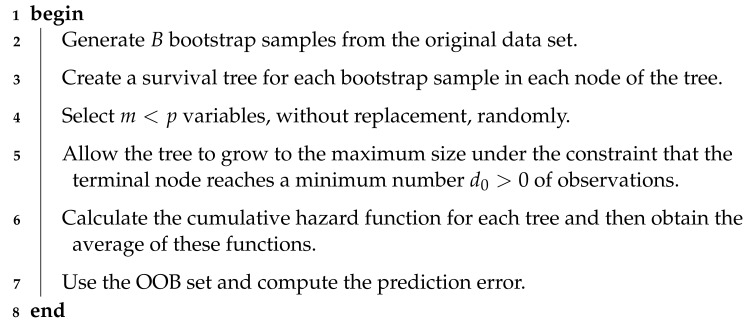


### 4.2. Node Splitting and Prediction

Recall the pair $\left(T_{i},\delta_{i}\right)$ is the death or censoring time of individual *i*, and $\delta_{i}=1$ if this individual experienced a death, and $\delta_{i}=0$ for a censoring, with i∈{1,…,n}. Also, recall $t_{1}<\ldots <t_{k}$ are *k* observed and ordered death times. The log-rank test for splitting a generic node *h* in child nodes $L=\{x_{i}\leq c\}$ (left) and $R=\{x_{i}>c\}$ (right), at the value *c* for a variable *x*, is given by

$$\begin{array}{c} \mathrm{LR}\left(x,c\right)=\frac{\sum_{h=1}^{k}\left(d_{h,L}-n_{h,L}d_{h}/n_{h}\right)}{\sqrt{\sum_{h=1}^{k}\left(n_{h,L}/n_{h}\right)\left(1-n_{h,L}/n_{h}\right)\left(\left(n_{h}-d_{h}\right)/\left(n_{h}-1\right)\right)d_{h}}}, \end{array}$$

where $n_{h,L}=\#\left\{T_{i}\geq t_{h},x_{i}\leq c\right\}$ and $n_{h,R}=\#\left\{T_{i}\geq t_{h},x_{i}>c\right\}$, with “#” denoting the cardinality of the specified set; and $x_{i}$ is the observed value of the variable *x* for the individual *i*, with i∈{1,…,n}. Thus, $n_{h,k}$ is the number of individuals at risk until $t_{j}$ in generic node *h* for child node *k*, with k∈{1(L),2(R)}, and $n_{h}=n_{h,L}+n_{h,R}$, that is, $n_{h}$ is the number of individuals in generic node *h*, where $n_{h,L}=\#\left\{i:x_{i}\leq c\right\}$ and $n_{h,R}=\#\left\{i:x_{i}>c\right\}$. In addition, $d_{h,k}$ is the number of deaths at $t_{j}$ in generic node *h* for child node *k*, where $d_{h}=d_{h,L}+d_{h,R}$, with h∈{1,…,k}.

The value |LR(x,c)| gives a measure of the separation of the nodes. As the value of |LR(x,c)| increases, the difference between the two child nodes increases, and the separation is better. In particular, the best split at node *h* is determined by finding the covariate $x^{*}$ and the split value $c^{*}$, so that $|\mathrm{LR}\left(x^{*},c^{*}\right)|\geq |\mathrm{LR}\left(x,c\right)|$ for all x,c. As the number of nodes increases and different cases are pushed apart, each node in the tree becomes homogeneous and is populated by cases with similar survival [18,44].

Using the Nelson–Aalen estimator described in Section 2.2 for the cumulative risk function of child node *k*, we have ${\hat{H}}_{k}\left(t\right)=\sum_{h:t_{h,k}\leq t}d_{h,k}/n_{h,k}$, where $d_{h,k}$, $n_{h,k}$ were defined previously, and $t_{h,k}$ is the observed death time at generic node *h* in child nodes *L* and *R*. Each individual *i* depends on a vector with *p* covariates $x_{i}^{⊤}$. Let $H(t\;|\;x_{i}^{⊤})$ be the cumulative hazard for individual *i*. To determine this value, we traverse $x_{i}^{⊤}$ in the tree, which falls on a single terminal node. Therefore, the cumulative hazard function for individual *i* is the Nelson–Aalen estimator for the terminal node of $x_{i}^{⊤}$, that is, $H\left(t\;|\;x_{i}^{⊤}\right)={\hat{H}}_{h}\left(t\right)$, for $x_{i}^{⊤}\in h$. If, at the end, there are *M* terminal nodes in the tree, then there are *M* estimates. Observe that all individuals within a given node have the same cumulative hazard function.

### 4.3. Cumulative Hazard Function for the OOB Set

Note that the cumulative hazard function described in $H(t\;|\;x_{i}^{⊤})$ is derived from a single tree. As we use bootstrap, it is coherent to consider $I_{i,b}=1$ if *i* is an individual of the OOB set for a given tree *b*. Otherwise, $I_{i,b}=0$. We can define $H_{b}^{*}\left(t\;|\;x_{i}^{⊤}\right)$ in the same way as $H(t\;|\;x_{i}^{⊤})$ for a tree grown from the *b*th bootstrap sample. The cumulative hazard function for individual *i* of the OOB set is given by $H_{e}^{**}\left(t\;|\;x_{i}^{⊤}\right)=\sum_{b=1}^{B}I_{i,b}H_{b}^{*}\left(t\;|\;x_{i}^{⊤}\right)/\sum_{b=1}^{B}I_{i,b}$. Notice that $H_{e}^{**}\left(t\;|\;x_{i}^{⊤}\right)$ is an average over bootstrap samples, where *i* is an individual belonging to the OOB set. In contrast to $H_{e}^{**}\left(t\;|\;x_{i}^{⊤}\right)$, we have the cumulative risk function of all individuals and not only those that belong to the OOB set, that is, $H_{e}^{*}\left(t\;|\;x_{i}^{⊤}\right)=\left(1/B\right)\sum_{b=1}^{B}H_{b}^{*}\left(t\;|\;x_{i}^{⊤}\right)$, where *B* is the number of bootstrap samples.

### 4.4. Prediction Error and Variable Importance

**Figure 2:**
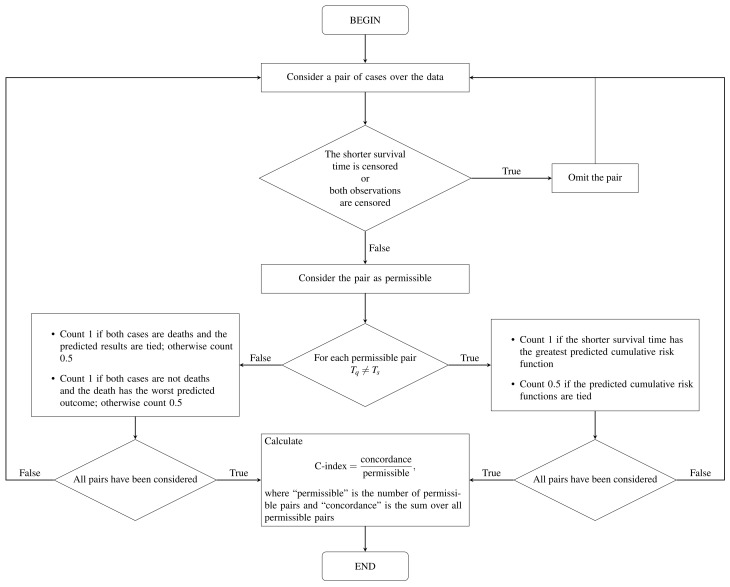
Flow diagram of the C-index computation based on [13].

To calculate the prediction error or the error rate of the OOB set, $H_{e}^{**}\left(t\;|\;x_{i}^{⊤}\right)$ is used as the cumulative hazard function for the individuals belonging to the OOB set. The prediction error is measured using the C-index [31]. To calculate this index, we need to define the worst predicted outcome. Let $t_{1}^{*}<\ldots <t_{k}^{*}$ be *k* observed and ordered death times. We say that individual *q* has a worse outcome than individual *s* if $\sum_{h=1}^{k}H_{e}^{**}\left(t_{h}^{*}\;|\;x_{q}^{⊤}\right)>\sum_{h=1}^{k}H_{e}^{**}\left(t_{h}^{*}\;|\;x_{s}^{⊤}\right).$ The C-index is calculated using the steps given in [18] and summarized in Figure 2.

We can select variables based on their importance, and using the OOB set. The procedure is performed as follows: (i) drop the OOB set onto the tree; and (ii) assign a random child node whenever a separation of the OOB set is stated. The VIMP of the OOB set is the prediction error for the original set subtracted from that of the new set obtained using random attributions of the OOB set. When the VIMP values are large, the variables have a predictive capacity. In contrast, values equal to zero or negative indicate non-predictive variables. It is incorrect to interpret the VIMP as an estimate of the change in the prediction error for a cultivated forest with and without a given variable. VIMP measures the difference in the prediction error of a new test case if the OOB set is unavailable, given that the original forest was cultivated using such data. However, in practice, this is often equal to the change in the prediction error for a cultivated forest with and without the OOB set, as the two quantities are conceptually different.

## 5. Application to Biomedical Data

In this section, we conduct the computational experiments with real data to illustrate our proposal. First, an exploratory data analysis is performed. Second, the results of the survival analysis are presented. Third, the results obtained by applying machine learning algorithms are provided.

### 5.1. Description of the Data Set and Exploratory Analysis

The data used in this study correspond to the Heart Institute, Hospital “das Clínicas”, Faculty of Medicine, University of São Paulo, Brazil, to compare the length of stay of n=145 patients with heart disease undergoing cardiac surgery [45]. The considered covariates are: age of the patient, type of protocol, race, sex, and type of patient. Let *T* be the response variable corresponding to the time between the entry and exit of the patient from the surgical ward in hours, whereas δ is an indicator variable of death or censoring. If δ=0, we have censoring; otherwise, we have a death. In the case of censoring, we do not have the exact information on the length of stay of this patient within the surgical ward, and, in the case of death, we have it exactly. Now, consider the following variables: the age of the hospitalized patient in years ($X_{1}$); the type of protocol ($X_{2}$), which can be conventional (0) or fast track (1); race ($X_{3}$), which is divided in white (1), black (2), and Asian (3); sex ($X_{4}$), divided between female (0) and male (1); and the type of patient ($X_{5}$) separated in congenital (0) and coronary (1). Regarding the variables sex and race, we do not know whether they were self-reported or designated by third parties and, concerning sex, if the classification was based on anatomical characteristics, genitalia, or self-reported. The fast-track protocol has, as its philosophy, a greater integration between the various teams that assist patients in reducing their length of stay, improving recovery, and reducing costs. Congenital heart disease was defined in [46] as a macroscopic structural abnormality of the heart or large intrathoracic vessels with relevant or potentially relevant functional repercussions. It is a condition that has existed since the fetal stage, appearing in the first eight weeks of pregnancy, when the organ is being formed. Coronary heart disease is caused by the accumulation of cholesterol in the arteries, which supplies the heart muscle.

**Figure 3:**
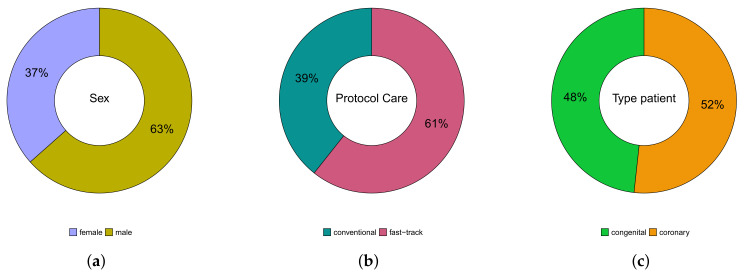
Pie charts of variables: gender (**a**), protocol care (**b**), and type of patient (**c**).

**Figure 4:**
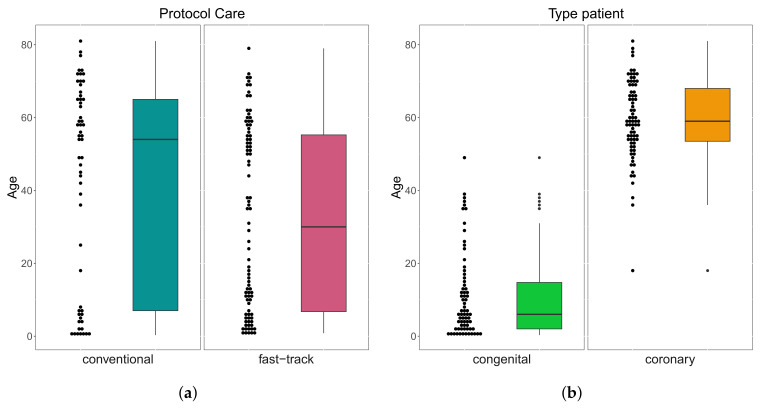
Box plots of age by protocol care (**a**) and type of patient (**b**).

A total of n=145 patients were followed up on, 53 (37%) were female, 92 (63%) were male; whereas their age varied between 3 months and 81 years; 138 (95%) were white, 4 (3%) black, and 3 (2%) Asian. Of the total number of patients, 57 (39%) were submitted to the conventional care protocol and 88 (61%) to the fast-track one. Furthermore, 70 (48%) have congenital heart disease and 75 (52%) have coronary heart disease. These results can be seen in Figure 3. In Table 1, we provide some descriptive measures for the ages (in years) of patients with both conditions and followed up in both protocols. Please note that the age of congenital heart disease patients who were followed up in the conventional protocol ranged from 3 months to 49 years, while, in the fast-track one, it went from 9 months to 38 years. For coronary heart disease patients, who were followed up in the protocol, it ranged from 18 to 81 years, and in the fast-track one, it went from 38 to 79 years. These results can be seen in Figure 4.

The absolute and relative sex distribution of patients with congenital and coronary heart disease followed up in the two protocols are shown in Table 2. We noticed that among the 53 (37%) female patients, 34 (64%) have congenital heart disease, 19 (36%) have coronary heart disease, 24 (45%) were followed up in conventional care protocol, and 29 (55%) in the fast-track one. Among the 92 (63%) male patients, 36 (39%) have congenital heart disease, 56 (61%) have coronary heart disease, 33 (36%) were followed up in the conventional care protocol, and  59 (64%) in the fast-track one.

The absolute and relative distribution by the race of patients with congenital and coronary heart disease followed up in both protocols are shown in Table 3.

We observed that among the 138 (95%) white patients, 69 (50%) have congenital heart disease, 69 (50%) have coronary heart disease, 52 (38%) were followed up in the conventional care protocol, and 86 (62%) in the fast-track one. Among the 4 (3%) black patients, 1 (20%) has congenital heart disease, 3 (80%) have coronary heart disease, 3 (75%) were followed up in the conventional protocol, and only 1 (25%) in the fast-track one. Furthermore, all patients of the Asian race have coronary heart disease, of which 2 (67%) were followed up in the conventional care protocol and 1 (33%) in the fast-track one.

### 5.2. Survival Analysis

**Figure 5:**
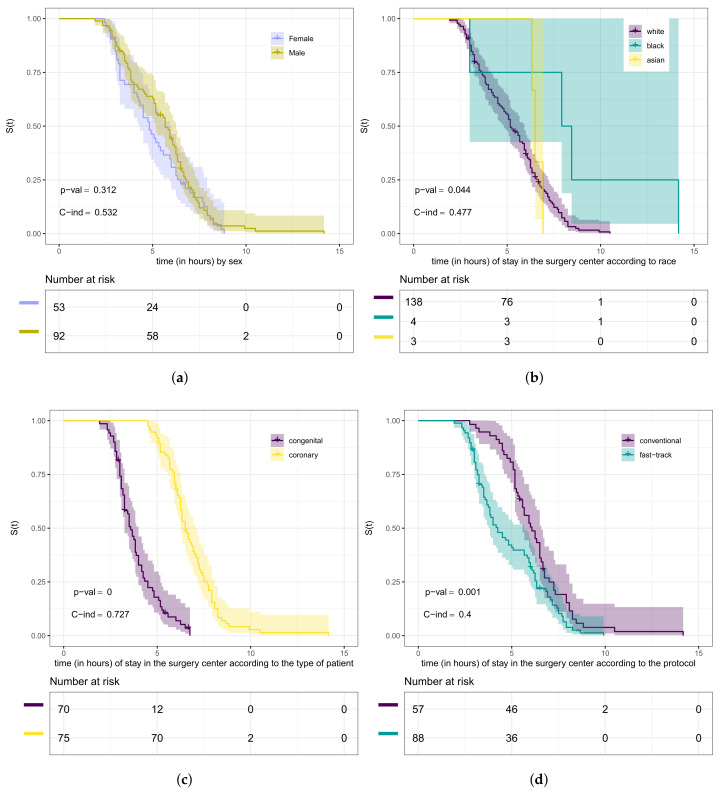
Kaplan–Meier curves S(t) (shading shows 95% CIs) with log-rank test, C-index, and risk tables for the variables: gender (**a**), race (**b**), type of patient (**c**), and type of protocol (**d**).

In Figure 5, we present the Kaplan–Meier curves for the variables sex, race, type of patient, and type of protocol according to the length of stay (in hours) in the surgical ward. From this figure, we can observe which curves are different. However, we need to conduct hypothesis tests to compare whether they differ significantly. We used in this step the log-rank test. The purpose of testing whether these curves are the same, in our case, is equivalent to testing whether the groups (strata) have the same length of stay (in hours) within the surgical ward. Therefore, our hypotheses are $H_{0}:$ “The lengths of stay of heart disease patients in the surgical ward are the same” versus $H_{1}$ being the negation of $H_{0}$.

The curves presented in Figure 5a provide evidence that the length of stay within the surgical ward of male patients has no difference compared to that of female ones. Through the test (*p*-value = 0.3), we can conclude that there is no significant difference at 5% in the length of stay according to sex. Furthermore, from Figure 5b, we can analyze it according to race. Using the log-rank test (*p*-value = 0.04), we concluded that the lengths of stay of these groups of patients are different. We detected the difference between white and black patients (*p*-value = 0.02) at the level of 5% of significance. Analyzing the curves in Figure 5c, we noticed evidence that there is a difference in the length of stay in the surgical ward concerning the type of patient.

Using the test (*p*-value < 0.0001), we could conclude that there is a significant difference at 5% for congenital and coronary patients. Then, we also verified, through the log-rank test (*p*-value = 0.001), that the curves presented in Figure 5d are significantly different at 5%, that is, the length of stay in the surgical ward according to the type of protocol is different. Here, we considered the Weibull regression model to verify if there is a relationship between the length of stay in the surgical ward and some covariates. It is worth mentioning that the exponential model was tested for this data set. However, no good fit was obtained compared to the Weibull model. In this step, we used the likelihood ratio test for nested models [47]. The maximum likelihood estimates, corresponding standard errors, and *p*-values for the hypothesis test of the significance of the parameters are presented in Table 4.

The Weibull model for our study is described as

$$\begin{array}{c} Y_{i}=\log\left(T_{i}\right)=\beta_{0}x_{i0}+\eta_{1}x_{i1}+\theta_{j}x_{i2}+\lambda_{k}x_{i3}+\mu_{l}x_{i4}+\rho_{m}x_{i5}+\sigma \nu_{i},\;i\in \left\{1,\ldots ,n\right\}, \end{array}$$

where each $Y_{i}$ is the logarithm of the length of stay of patient *i* in the surgical ward, with j,l,m∈{1,2}, k∈{1,2,3} and $\nu_{i}=\log\left(\varepsilon_{i}\right)$. As we assume a reference case parameterization, we have the constraints $\theta_{1}=0$, $\lambda_{1}=0$, $\mu_{1}=0$, and $\rho_{1}=0$.

We consider a significance level of 5% to select which variables should be included in the model. We noticed that the variable’s type of the protocol, race, and type of patient are significant at 5% for the model. In Table 5, we present the maximum likelihood estimates,  corresponding standard errors, and *p*-values for the selected variables.

**Figure 6:**
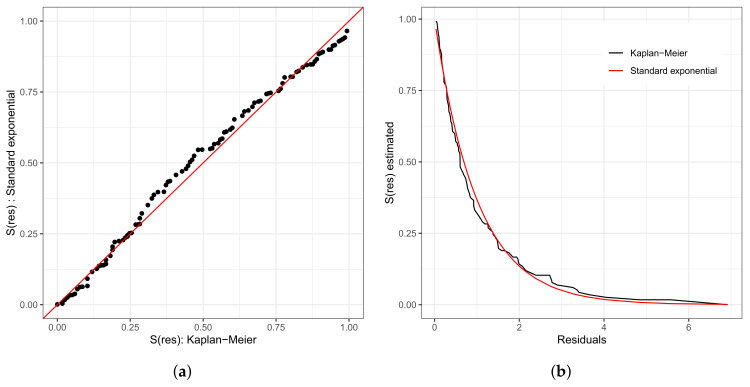

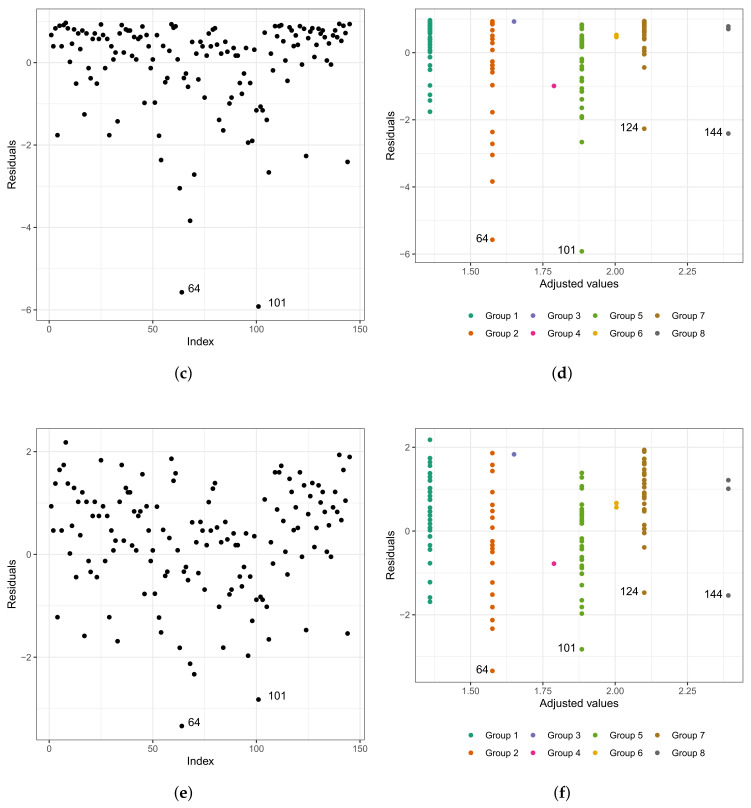
Plots of Cox–Snell (**a**,**b**), martingale (**c**,**d**), and deviance (**e**,**f**) residuals of the Weibull regression model adjusted to the data of length of stay in the surgery ward.

As in any other statistical model, evaluating the fitted model is very important. To investigate the fit of the fitted Weibull regression model, we use residual analysis. In Figure 6a,b, we present the Cox–Snell residuals of the Weibull regression model, adjusted to the data set referring to the length of stay within the surgical ward of congenital and coronary heart disease patients submitted to cardiac surgery in the fast-track protocol compared to the conventional one. We observed that the Weibull regression model is acceptable for the residuals. Therefore, it presented a satisfactory fit to the data on the length of stay. To verify the existence of potentially influential observations, we show in Figure 6c–f the graphs of the martingale and deviance residuals against the indices of individuals and adjusted values, respectively. As we can see in the figures mentioned above, four observations stand out as potentially influential points, namely: #64, #101, #124, and #144. In Figure 6b,c, we noticed a random behavior of the residuals, with an emphasis only on points #64 and #101.

We can see in the graph that the points #64 and #101 are further away from the other ones. Observing Figure 6e,f, we noticed that the patients are divided into “eight” groups with common characteristics among themselves, that is, we have the following:

(i) Group 1: white and congenital patients in the fast-track care protocol.
(ii) Group 2: white and congenital patients in the conventional care protocol. Here, the highlight is for patient #64, 1 year old, female, and the time spent in the surgical ward is longer than 6.67 h; that is, the exact time is unknown. In this group, the longest stay in the surgical ward is that of patient #68 (6.75 h), aged 0.6 (approximately 7 months), and female. The highest age for this group is 49 years, and the minimum is 0.3 (approximately 3 and a half months). The youngest is patient #58, with 4.50 h of length of stay.
(iii) Group 3: patients in the fast-track care protocol, black, and congenital (only patient #25).
(iv) Group 4: patients in the fast-track care protocol, Asian, and coronary (only patient #87).
(v) Group 5: patients in the fast-track care protocol, white and coronary. Here, we highlight patient #101, aged 60 years, male, and 9.92 h of length of stay. In this group, patient #101 is the one with the longest length of stay. Patient #88 has the same characteristics as patient #101. However, their length of stay is 7.50 h, approximately 24% less than that of patient #101.
(vi) Group 6: patients in the conventional care protocol, Asian, and coronary (patients #135 and #141).
(vii) Group 7: patients in the conventional care protocol, white and coronary. The highlight here is for patient #124, aged 60 years, male, and with 10.50 h of length of stay. In this group, patient #124 has the longest length of stay. The oldest patient in this group is 81 years old, male, with 7.33 h of length of stay.
(viii) Group 8: patients in the conventional care protocol, black, and coronary (patients #131, #132, and #144). Patient #131 is 58 years old, male, and with 8.45 h of length of stay; patient #132 is 47 years old, male, and with 7.92 h of length of stay; and patient #144 is 59 years old, male, and with 14.17 h of length of stay. We must highlight that patient #144, even with close similarities to patient #131, had a longer stay of 40% than the latter.

To analyze the impact of the highlighted points in the parameter estimates, we performed a confirmatory analysis by readjusting the model, eliminating individually and jointly the potentially influential observations. In Table 6, we report the maximum likelihood estimates and the respective *p*-values for the model parameters in parentheses.

Through the confirmatory analysis, we observed that the variable race is not significant for the model at 5%. Note that when we remove point #144 and all the points together (#64,#101, #124, and #144), considered as potentially influential, the significance of the variable changes, that is, these influential points were masking its significance. The other variables are still significant at 5% for the model with *p*-values smaller than 0.0001 (0.01%). Therefore, we proceed with the analysis without the variable sex. Every previous process is repeated considering only the variables type of protocol and type of patient.

In Table 7, we present the maximum likelihood estimates, corresponding standard errors, and *p*-values for the model without the variable race. We noticed that all variables are significant at the usual levels, that is, 5% and >10%.

**Figure 7:**
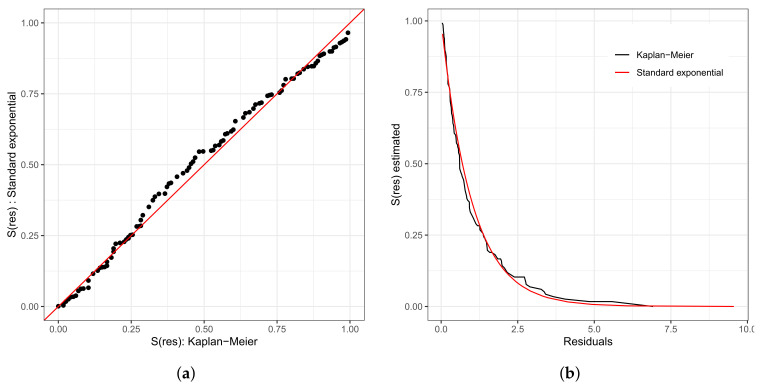

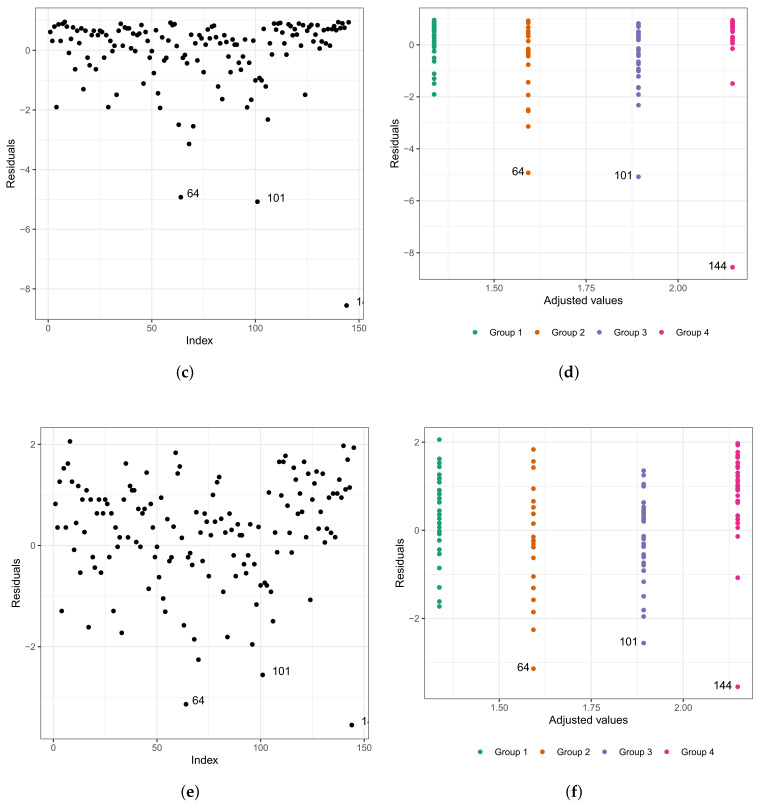
Plots of Cox–Snell (**a**,**b**), martingale (**c**,**d**), and deviance (**e**,**f**) residuals of the Weibull regression model after model selection adjusted to the data of length of stay in the surgery ward.

We repeat the process of evaluating the refitted model; that is, we used the analysis of the residuals. In Figure 7a,b, we present the Cox–Snell residuals of the Weibull regression model adjusted to the data of the length of stay in the surgical ward. We observed indications that this model is acceptable for the residuals. Therefore, it presented a satisfactory fit to these data. To verify the existence of potentially influential observations, we present in Figure 7c–f the graphs of the martingale and deviance residuals against the indices of individuals and adjusted values, respectively. Unlike Figure 6c–f, here we have three observations that stand out as potentially influential points, namely: #64, #101, and #144. They have all been previously described.

It is important to highlight that considering the final fitted model, that is, the model that selected only the variables type of protocol and type of patient, the patients are now divided into four groups with common characteristics among themselves. Thus, we have the following:

(i) Group 1: congenital patients in the fast-track care protocol.
(ii) Group 2: congenital patients in the conventional care protocol.
(iii) Group 3: coronary patients in the fast-track protocol.
(iv) Group 4: coronary patients in the conventional care protocol.

This justifies the fact that the points are not dispersed in the graphs of the residuals presented in Figure 7e,f, since within each group, the patients present similar characteristics. Observation #64 corresponds to the individual with a longer stay in the surgical ward than 75% of the other patients (6.67 h, we do not know the exact length of stay, only that it is greater than 6.67 h).

Using the adjusted model to predict the length of stay inside the surgical ward, this value is approximately 4.92. Calculating the values of martingale and deviance residuals, we have the following results: −4.93 and −3.14, respectively. Observation #101 corresponds to the individual with the third-longest length of stay in the surgical ward (9.92 h). However, if we use the fitted model to predict its length of stay, this value is approximately 6.63 h. Calculating the values of the martingale and deviance residuals, we have the following results: −5.08 and −2.56, respectively. In addition, observation #144 corresponds to the individual with the longest length (14.17 h). Nevertheless, if we use the fitted model to predict its length of stay, this value is approximately 8.57 h (the estimated time corresponds to 60.47% of the observed time). Calculating the values of the martingale and deviance residuals, we have the following results: −8.57 and −3.55, respectively.

We analyze the impact of observations #64, #101, and #144 on the parameter estimates, performing a confirmatory analysis readjusting the model, eliminating individually and jointly the observations detected as potentially influential. Table 8, reports the corresponding estimates and *p*-values (in parentheses). By the confirmatory analysis, we observed that the highlighted points do not change the significance of the variables selected for the model.

With the fitted model, we can interpret the estimated coefficients presented in Table 5. The direct interpretation, as performed in linear regression, is not possible in this context since the scale of the response was transformed to a logarithmic one. An interesting interpretation is the ratio of median times [48], which compares the median survival time between groups. Therefore, we can compare the median length of stay in the surgical ward for patients under the fast-track care protocol with those under the conventional one, as well as the median length of stay of congenital patients with the coronary ones. Note that the median length of stay of patients in the fast-track care protocol was reduced by approximately 77.4% when compared to those under the conventional one. Furthermore, the median length of stay of coronary patients within the surgical ward was approximately 1.7 times greater than that of congenital patients. To finalize the fitting of the model, we calculated the C-index, which resulted in 0.76, implying a prediction error of 23.85%.

### 5.3. Analysis Using Machine Learning Algorithms

Another alternative to analyzing these data is to use the RSF method. We employed the randomForestSRC package that has implementations of various survival cases that come from the randomSurvivalForest package [49] for regression and classification, as well as multivariate and unsupervised forests.

We simulated 1000 random survival trees and tested three variables for each randomly chosen node ($\sqrt{p}$, with *p* being the number of covariates, the final value is rounded to the next integer value). Each split was made using the log-rank separation, as discussed in Section 4.2, and the minimum size of the selected terminal node was ten. The analysis was realized using the R software, version 4.2.2, for the Linux operating system.

**Figure 8:**
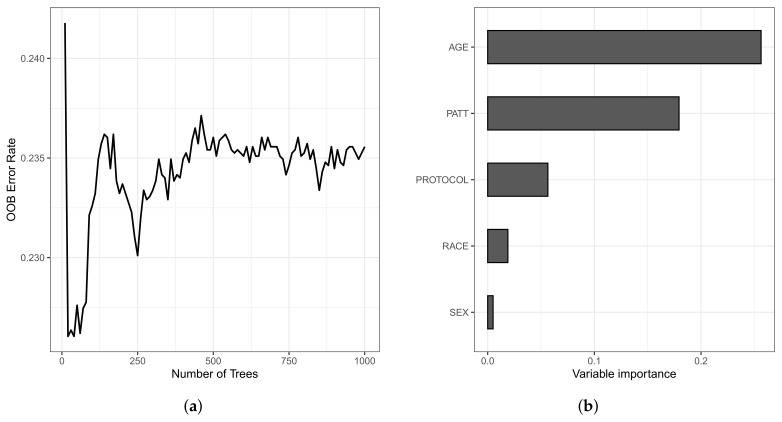
Plots of the OOB set error rate (**a**) and VIMP measure of the variable (**b**).

The OOB set is the error rate for the trained model, applied to data not included in the training set of a specific tree. The model presented an error rate of 23.55% for the training data and 20.31% for the testing data. Still, the C-index for the OOB set was 0.7644, and 0.7969 for the training and testing data sets, respectively. In Figure 8, we present the OOB set error rate and the VIMP measures. Note that, in Figure 8a, from 400 trees, the error rate stabilizes around 0.235 (23.50%). In Figure 8b, we present the VIMP measures, whose variables are the age of the patient (in years) at admission (AGE); type of protocol (PROTOCOL); race (RACE); sex (SEX); and type of patient (PATT).

All variables have positive VIMP values, with the variables age, type of patient, and type of protocol having higher VIMP (0.2597, 0.1822, and 0.0556, respectively), indicating the predictive power of the RSF method is dependent on these variables. We also noticed that, in the Weibull regression model, the age variable was non-significant at 5%. Then, this variable was excluded of the model from the beginning of the analysis. Nonetheless, in a survival forest, this variable is the most important since it has the highest VIMP, a value of 0.2597.

Table 9 shows a summary of the comparative analysis between the models, considering error rate, C-index, and the most predictive model variables.

## 6. Discussion and Conclusions

We observed a reduction in hospital stay for patients undergoing fast-track protocol compared to conventional protocol, resulting in decreased professional occupation time and costs for the institution, as reported in [50]. Additionally, this approach increases bed availability for new patients. The fast-track protocol is also being adopted in other surgeries successfully [51].

Out of n=145 patients, 88 were assisted by the fast-track protocol group regardless of their heart disease, representing 61% of the patients. The longest stay in the surgical center within this group was 9.92 h for a 60-year-old patient with coronary heart disease. In the conventional protocol group, the longest stay was 14.17 h for a 59-year-old patient with coronary heart disease. The shortest stay in the fast-track group was 1.92 h for a 2-year-old patient with congenital heart disease, while in the conventional protocol group, the shortest stay was 2.75 h for a 1-year-old patient with congenital heart disease. These statistics reinforce one of the benefits of adopting this protocol. Moreover, the average hospital stay for patients assisted by the fast-track protocol was shorter than for those under the conventional protocol, with 4.773 h compared to 6.188 h, respectively. Another noteworthy point is the age variability of patients assisted by the fast-track protocol, ranging from 8.3 months to 79 years old.

Possible limitations include the availability of this type of protocol in hospitals, as it usually requires more infrastructure, as well as the adherence of medical teams to this protocol, as they often prefer the conventional protocol due to their experience. Therefore, it is essential to produce and disseminate studies that prove the efficiency and benefits of new protocols in healthcare while not disregarding the efficacy and use of the usual protocol. The idea is to have an additional protocol available, rather than a replacement for the conventional one.

Random forest has limitations such as long computing time in large data sets, non-generalizability, and difficulty in clinical interpretation. Furthermore, when using parametric models such as the Weibull regression, certain assumptions must be met, such as the hazard rate changing over time and the absence of outliers, which can affect the accuracy of the estimates as observed in this study.

If a practitioner also wants an interpretable model, choosing the method depends on the specific characteristics of the data and the goals of the analysis. Random forest and Weibull models serve different purposes, and each has its advantages and limitations. If the practitioner values interpretability and has prior knowledge of the underlying distribution of survival times, the Weibull model may be more suitable. Nonetheless, if the practitioner prioritizes predictive accuracy and has no prior assumptions about the distribution of survival times, random forest may be more appropriate. In any case, both methods are powerful tools for predicting outcomes.

In this work, we used three criteria to carry out a comparative study between Weibull regression and random forest models when analyzing survival data: (i) error rate, (ii) Harrell C-index, and (iii) selection of appropriate variables for survival analysis. In the computational experiments, we employed the R software, in particular the randomForestSRC package. The used data belong to the Heart Institute of the Hospital “das Clínicas”, in Brazil, and correspond to patients who underwent cardiac surgery. The models analyzed respond in a very similar way, with only slight differences.

Although the lowest error rate was obtained with the random forest model, the Weibull regression reached a higher C-index. Both models agree that variables type of patient and type of protocol are the most predictive, but only the random forest model considers the variable age. We believe that the comparative study that we propose in this study is relevant and can be used by many medical researchers to analyze their survival data.

Survival analysis is not only important in medicine, as in biology, it has many applications. For biologists, the study of microorganisms is of particular interest. Some microorganisms favor the life of plants, animals, and people. Nevertheless, there are also pathogenic microorganisms. Therefore, studying the lifetime of microorganisms is essential [52,53]. For example, it is important to study the survival of viruses and bacteria that affect humans [54]. In the case of viruses, SARS-Cov2 has been of great interest to researchers for the last three years [55]. In a recent investigation, a survival analysis was performed on COVID-19 patients [56]. In [57], we can see the use of fuzzy logic and artificial intelligence techniques for the remote monitoring of cardiac arrhythmia in COVID-19 patients. In telemetry, it is important to pay more attention to those patients who fell ill before and who are at higher risk of becoming ill again [58]. The survival study plays a fundamental role in patient telemetry. Fungi are also entities of interest to biologists and doctors. In [59,60], we can see research that uses survival analysis to study certain types of fungi. All the aforementioned applications offer the opportunity to be applied to our proposal. For this reason, in a future work, we are interested in building an R package that facilitates the comparative study that we propose, in such a way that it can be applied by any researcher to other data sets.

## Figures and Tables

**Table 1:** Descriptive measures for the ages (in years) of patients with congenital and coronary heart disease followed up in both protocols.

Age	Congenital		Coronary	
	Conventional Care	Fast-Track Care	Conventional Care	Fast-Track Care
Total (n=145)	20	50	37	38
Mean	8.5	12.2	60.5	58.4
Standard deviation	13.4	11.1	12.9	8.8
Minimum	0.3	0.8	18.0	38.0
Median	4.0	10.0	63.0	58.0
Maximum	49.0	38.0	81.0	79.0

**Table 2:** Absolute and relative distribution for sex of patients with congenital and coronary heart disease followed up in both protocols.

Heart Disease	Care Protocol	Sex			
		Female	%	Male	%
Congenital	Conventional Care	13	65	7	35
	Fast-track Care	21	42	29	58
Coronary	Conventional Care	11	30	26	70
	Fast-track Care	8	21	30	80
Total		53	37	92	63

**Table 3:** Absolute and relative distribution for race of patients with congenital and coronary heart disease followed up in both protocols.

Heart Disease	Care Protocol	Race					
		White	%	Black	%	Yellow	%
Congenital	Conventional Care	20	100	0	0	0	0
	Fast-track Care	49	98	1	2	0	0
Coronary	Conventional Care	32	87	3	8	2	5
	Fast-track Care	37	97	0	0	1	3
Total		138	95	4	3	3	2

**Table 4:** Weibull regression model parameter estimates (full model).

Parameter	Covariates	Estimate	Standard Error	*p*-Value
$\beta_{0}$	intercept	1.6020	0.0412	<0.0001
$\eta_{1}$	age	−0.0004	0.0017	0.8110
$\theta_{2}$	type of protocol - fast-track care	−0.2065	0.0392	<0.0001
$\lambda_{2}$	race - black	0.3125	0.1123	0.0054
$\lambda_{3}$	race - Asian	−0.0918	0.1244	0.4606
$\mu_{2}$	sex - male	−0.0583	0.0399	0.1433
$\rho_{2}$	type of patient - coronary	0.5599	0.0931	<0.0001
$\tau^{a}$	-	−1.5626	0.0637	<0.0001
$\sigma^{b}$	-	0.2100	-	-
$\gamma^{c}$	-	4.5872	-	-

where *a*: scale parameter logarithm, *b*: scale parameter, and *c*: shape parameter.

**Table 5:** Weibull regression model parameter estimates (after model selection).

Parameter	Covariates	Estimate	Standard Error	*p*-Value
$\beta_{0}$	intercept	1.5775	0.0330	<0.0001
$\theta_{2}$	type of protocol - fast-track	−0.2158	0.0391	<0.0001
$\lambda_{2}$	race - black	0.2907	0.1127	0.0099
$\lambda_{3}$	race - Asian	−0.0963	0.1252	0.4414
$\rho_{2}$	type of patient - coronary	0.5250	0.0382	<0.0001
$\tau^{a}$	-	−1.5517	0.0634	<0.0001
$\sigma^{b}$	-	0.2120	-	-
$\gamma^{c}$	-	4.7170	-	-

where *a*: scale parameter logarithm, *b*: scale parameter, and *c*: shape parameter.

**Table 6:** Maximum likelihood estimates and *p*-values in parentheses for parameters $\theta_{2}$, $\lambda_{2}$, $\lambda_{3}$, and $\rho_{2}$ of the Weibull regression model after removing the points.

Removed Observation	Parameter Estimates				
	$\beta_{0}$	$\theta_{2}$	$\lambda_{2}$	$\lambda_{3}$	$\rho_{2}$
#64	1.5538 (<0.0001)	−0.1995 (<0.0001)	0.3046 (0.0057)	−0.0958 (0.4347)	0.5364 (<0.0001)
#101	1.5856 (<0.0001)	−0.2291 (<0.0001)	0.3053 (0.0048)	−0.0077 (0.5175)	0.5074 (<0.0001)
#124	1.5693 (<0.0001)	−0.2024 (<0.0001)	0.3085 (0.0063)	−0.0872 (0.4850)	0.5134 (<0.0001)
#144	1.5776 (<0.0001)	−0.2140 (<0.0001)	−0.0535 (0.6700)	−0.0962 (0.4300)	0.5227 (<0.0001)
{#64,#101, #124, #144}	1.5605 (<0.0001)	−0.1971 (<0.0001)	−0.0180 (0.8800)	−0.0668 (0.5600)	0.5027 (<0.0001)

**Table 7:** Weibull regression model parameter estimates.

Parameter	Covariates	Estimate	Standard Error	*p*-Value
$\beta_{0}$	intercept	1.5928	0.0356	<0.001
$\theta_{2}$	type of protocol - fast-track care	−0.2557	0.0396	<0.001
$\mu_{2}$	type of patient - coronary	0.5552	0.0383	<0.001
$\tau^{a}$	-	−1.5010	0.0621	<0.001
$\sigma^{b}$	-	0.2230	-	-
$\gamma^{c}$	-	4.4843	-	-

where *a*: scale parameter logarithm, *b*: scale parameter, and *c*: shape parameter.

**Table 8:** Maximum likelihood estimates (all the *p*-values are <0.001) for parameters $\beta_{0}$, $\theta_{2}$, and $\rho_{2}$ of the Weibull regression model after removing the points.

Removed Observation	Parameter Estimates		
	$\beta_{0}$	$\theta_{2}$	$\rho_{2}$
#64	1.5720	−0.2414	0.5683
#101	1.6036	−0.2713	0.5412
#144	−1.5753	−0.2107	0.5172
{#64,#101, #144}	1.5658	−0.2103	0.5120

**Table 9:** Summary of the comparative study between the RSF and the Weibull regression models.

Model	Error Rate	C-Index	Most Predictive Variables
RSF (training data)	23.55%	0.76	age, type of patient, type of protocol
RSF (testing data)	20.31%	0.79	age, type of patient, type of protocol
Weibull regression	23.82%	0.76	type of patient, type of protocol

## Data Availability

To ensure transparency and reproducibility [61], the data and codes used in this study are available at https://github.com/Raydonal/ML-Weibull (accessed on 7 March 2023).
